# Amlodipine + atorvastatin single pill is the most effective choice for primary prevention

**Published:** 2010-08

**Authors:** J Aalbers

**Affiliations:** Special Assignments Editor

## Introduction

Preventing cardiovascular events in at-risk patients is greatly enhanced if a single pill is prescribed for the commonly occurring risks of hypertension and dyslipidaemia

In this new study using data from a commercially managed healthcare organisation in the United States, only one in five patients adhered to and took their preventative tablets as medication for dyslipidaemia and hypertension if they were taken as two individual tablets. If the medication was given as a single tablet, in this case atorvastatin plus amlodipine, adherence improved to one in two patients.

This 150% increase in compliance is very significant and applies to everyday clinical practice, compared to lower compliance differences between single pills and two or more pills, which was found in specially designed compliance studies.

The overall benefit in terms of the reduction in cardiovascular events is substantiated in this study, which showed similar low rates (1.88 per 100 personyears) for patients who were adherent to their medication (whether one pill or two), compared to 2.47 events per 100 person-years in non-adherent patients.

In essence, if the single pill increases compliance two-fold and compliance reduces events by 30% compared to noncompliance, the role of the single pill in primary prevention is the most effective choice for commercially managed healthcare funds.

The evaluation of the US commercially managed healthcare system included patients with hypertension and dyslipidaemia but without diabetes, and evaluated the cardiovascular events in adherent patients in categories of either two pills or the single-pill calcium channel blocker (SPAA) and a statin.

The primary outcome measure was the rate of cardiovascular events occurring in the six- to 18-month period following the index date, set as the date when the patient was started on either the SPAA or the two-drug, two-pill regimen. Patients who had a cardiovascular event in the preceding six months were excluded from the analysis.

Adherence levels were much higher for the single-pill approach (Table 1).

**Table 1:** ADHERENCE MEASURES FOR SPAA AND CCB + STATIN (TWO PILLS) IN PRIMARY-PREVENTION PATIE

**	*SPAA (n = 1 537)*		*CCB + statin (n = 17 910)*		*p-value*
Six months					
Number (%) of patients with PDC ≥ 80%	868	56.5%	3 825	21.4%	< 0.001
Mean PDC (SD)	0.73	(0.26)	0.49	(0.31)	
Median PDC	0.83		0.50		< 0.001
12 months					
Number (%) of patients with PDC ≥ 80%	712	46.3%	3 529	19.7%	< 0.001
Mean PDC (SD)	0.66	(0.30)	0.46	(0.31)	
Median PDC	0.75		0.46		< 0.001
18 months					
Number (%) of patients with PDC ≥ 80%	650	42.3%	3 342	18.7%	< 0.001
Mean PDC (SD)	0.62	(0.31)	0.43	(0.32)	
Median PDC	0.72		0.42		< 0.001

Importantly, the reduction in cardiovascular events in adherent patients was the same regardless of whether patients were on a single- or two-pill treatment regimen.

**Table 2:** CARDIOVASCULAR EVENTS FROM SIX MONTHS FOLLOWING INITIATION OF SPAA OR CCB + STATIN IN PRIMARY-PREVENTION PATIENTS

**	*Overall (n = 19 447)*	*Adherent (n = 4 693)*	*Nonadherent (n = 14 754)*	*SPAA (n = 1 537)*	*CCB + statin (n = 17 910)*
*12-month event rate*					
*Total events (n)*	*452*	*88*	*364*	*19*	*433*
*Total person-years*	*19 447*	*4 693*	*14 754*	*1 537*	*17 910*
*Incidence rate per 100 person-years*	*2.32*	*1.88*	*2.47*	*1.24*	*2.42*
*Overall event rate*					
*Total events (n)*	*818*	*164*	*654*	*38*	*780*
*Total person-years*	*38 074*	*9 139*	*28 935*	*2 734*	*35 340*
*Incidence rate per 100 person-years*	*2.15*	*1.79*	*2.26*	*1.39*	*2.21*
